# 吸烟对男性肺癌患者组织学分型的影响及其趋势分析

**DOI:** 10.3779/j.issn.1009-3419.2017.08.03

**Published:** 2017-08-20

**Authors:** 漫漫 贾, 纪宾 李, 华 林, 小农 邹, 平 赵

**Affiliations:** 100021 北京，国家癌症中心/中国医学科学院北京协和医学院肿瘤医院，全国肿瘤防治研究办公室 National Cancer Center/Cancer Hospital, Chinese Academy of Medical Sciences and Peking Union Medical College, Beijing 100021, China

**Keywords:** 肺肿瘤, 吸烟史, 组织学, 流行病学, Lung neoplasms, Smoking history, Histology, Epidemiology

## Abstract

**背景与目的:**

已有的研究发现我国肺癌患者的组织学亚型分布有变化。本研究旨在探讨吸烟对我国男性肺癌患者组织学分型的影响及变化趋势。

**方法:**

收集和整理2000年-2012年中国医学科学院肿瘤医院诊治的男性肺癌患者人口学、吸烟史、组织学等信息，用年度百分比变化（annual percentage change, APC）进行趋势检验。

**结果:**

入选肺癌14, 106例，其中吸烟11, 750例，不吸烟2, 356例。吸烟患者中，鳞癌的比例为39.38%，腺癌29.85%；2000年-2012年，鳞癌比例从44.19%下降至35.50%（APC=-1.9%, *P* < 0.001）；腺癌从15.25%上升至41.85%（APC=6.8%, *P* < 0.001）；腺鳞癌从4.13%降低至0.72%（APC=-14.9%, *P* < 0.001）。不吸烟患者中，腺癌53.86%，鳞癌16.64%；腺癌从38.03%上升至67.83%（APC=4.3%, *P* < 0.001）；大细胞癌和腺鳞癌呈波动变化。

**结论:**

肺腺癌在男性吸烟和非吸烟肺癌患者中的比例均显著上升，其非吸烟因素暴露与肺癌的关系应进一步深入研究。

## Abstract (en)

**Background and objective:**

Studies found that there were changes in histological subtypes of lung cancer patients in China. This study investigated the effect of smoking on lung cancer histology and its trend in Chinese male.

**Methods:**

Demographic, smoking history and histological information about male lung cancer patients diagnosed or treated from 2000 to 2012 was collected from Cancer Hospital, Chinese Academy of Medical Science. Trends of histological subtypes calculated with annual percentage change (APC).

**Results:**

A total of 14, 106 patients with lung cancer were enrolled, with smoking 11, 750 cases and non-smoking 2, 356 cases. The main histological type of smoking lung cancer was squamous cell carcinoma (SCC)(39.38%), followed by adenocarcinoma (ADC)(29.85%). Among smokers, the proportion of SCC decreased from 44.19% to 35.50% (APC=-1.9%, *P* < 0.001), however, the ADC increased from 15.25% to 41.85% (APC=6.8%, *P* < 0.001). Adenosquamous carcinoma (ASC) was from 4.13% to 0.72% (APC=-14.9%, *P* < 0.001). In non-smokers, the ADC was 53.86%, and SCC was 16.64%. ADC increased from 38.03% to 67.83% (APC=4.3%, *P* < 0.001). Distributions of LCC and ASC were scattered.

**Conclusion:**

Proportion of ADC increased significantly in smoking and non-smoking lung cancer patients, and the relationship between non-smoking factor exposure and lung cancer should be further studied.

据国家癌症中心报告，2013年我国男性肺癌世标发病率为49.62/10万，世标死亡率为40.30/10万，均位居恶性肿瘤首位^[1]^。全国三次死因调查数据显示，近30年中国男性肺癌死亡率持续快速上升（世标率：1973年-1975年：10.10/10万，1990年-1992年：29.70/10万，2004年-2005年：39.06/10万）^[2]^。国内外研究^[3-5]^显示，近年内肺癌病理类型也发生了变化，以肺腺癌上升和肺鳞癌下降为主要趋势。吸烟可导致肺癌的发生，同时也可能是肺癌病理类型变化的影响因素^[6, 7]^。我国男性吸烟率（62.8%）远高于女性（3.1%）^[8]^，是男性肺癌的主要危险因素，故本文以北京某三甲医院诊治的男性肺癌患者为研究对象，探讨中国人群中吸烟和不吸烟男性肺癌患者的组织学特征及变化趋势。

## 1 资料与方法

### 1.1 资料来源

本研究收集2000年1月1日-2012年12月31日中国医学科学院肿瘤医院诊治的男性肺癌患者（ICD10:C34）的人口学信息（性别、出生日期、居住地）、解剖学和组织学信息和吸烟史信息（吸烟情况、吸烟年限、日吸烟量）。根据2010年全球成人烟草调查方法，将有吸烟史者定义为吸烟者^[8]^。排除重复和年龄、吸烟史、组织学信息缺失者后，共入选14, 106例。

### 1.2 组织学亚型分类

根据文献报道，将肺癌组织学亚型分类为以下6类：鳞癌、腺癌、小细胞肺癌、大细胞肺癌、腺鳞癌和其他^[9]^。

### 1.3 统计学方法

统计学分析采用SAS 9.3和Joinpoint 4.3.1.0。计算吸烟和非吸烟肺癌患者的年龄、时期、组织学亚型和地区分布差异采用*χ*^2^检验。肺癌组织学亚型趋势变化用年度百分比变化（annual percentage change, APC）表示，利用Joinpoint软件模型进行计算，统计学差异性检验采用*Z*检验。以*P* < 0.05为差异有统计学意义。

## 2 结果

### 2.1 基本情况

研究对象中，以60岁-69岁为主（4, 641例，占32.90%）；2000年肺癌患者为458例，占3.25%；2012年1, 531例，占10.85%；2011年达到高峰（12.97%）；组织学亚型以鳞癌、腺癌、小细胞癌为主；居住地以北京最多，为4, 247例，占30.11%，其次为河北（16.34%）、内蒙古（8.93%）。

吸烟的肺癌患者为11, 750例（83.30%），不吸烟患者2, 356例（16.70%）；30岁及以上吸烟患者比例是不吸烟的2倍以上。有吸烟史患者的比例在肺鳞癌最高，为92.19%，其次为小细胞癌（86.96%），腺癌最低，为73.43%。

### 2.2 肺癌吸烟患者的组织亚型变化

2000年，肺癌吸烟患者中鳞癌所占比例为44.19%，2012年为35.50%（APC=-1.9%, *P* < 0.001）；腺癌从15.25%上升至41.85%（APC=6.8%, *P* < 0.001）；大细胞癌在2000年-2009年上升迅速（APC=15.0%, *P*=0.01），之后呈下降趋势（APC=-16.2%, *P*=0.09）；腺鳞癌从4.13%下降至0.72%（APC=-14.9%, *P* < 0.001）。小细胞癌较为稳定（APC=1.5%, *P*=0.23）。肺癌的其他组织学型别呈下降趋势（APC=-10.6%, *P* < 0.001）。

### 2.3 肺癌不吸烟患者组织亚型变化趋势

肺癌不吸烟患者中腺癌所占比例每年以4.3%速度上升，从38.03%上升至67.83%（*P* < 0.001）。鳞癌从23.94%下降至13.99%，每年以3.0%速度下降，但未发现该下降具有统计学意义（*P*=0.07）；小细胞癌从15.49%下降至8.04%（APC=-0.6%, *P*=0.78）；大细胞癌和腺鳞癌所占比例呈波动分布。肺癌其他组织学型别从21.13%下降至5.24%，每年以10.7%速度下降（*P* < 0.001）。

### 2.4 吸烟量与肺癌患者主要组织亚型变化

肺癌吸烟患者中，吸烟20包年及以上者的鳞癌所占比例（44.41%）高于腺癌（27.34%）和小细胞癌（13.86%）。随着吸烟剂量的上升，小细胞癌所占比例下降（18.88%, 13.54%, 13.86%），腺鳞癌上升（1.77%, 1.99%, 2.11%）。

2000年-2012年，吸烟 < 10包年的鳞癌下降速度（APC=-3.3, *P* < 0.001）高于吸烟≥20包年者（APC=-1.3, *P* < 0.001），但腺癌上升速度则低于吸烟≥20包年者（APC=6.5, 7.1; *P* < 0.001）。小细胞癌中，吸烟10包年以下者每年以3.6%的速度显著上升，但吸烟10包年-19包年者则每年以5.0%的速度显著下降。

## 3 讨论

70%的肺癌死亡可归因于吸烟^[10]^。本研究数据显示，男性肺癌患者83.30%吸烟，16.70%不吸烟。烟草烟雾中含有多种致癌物，其中多环芳香烃类、N-亚硝胺类等均可诱发呼吸道癌症的发生^[11]^。国内外研究^[12-14]^显示，吸烟与肺鳞癌关系尤为密切，随着吸烟剂量的上升而增强，非吸烟者中肺腺癌占有主要地位。中国男性吸烟率仍居高不下，但研究^[15, 16]^显示，近年来中国人群的肺癌组织学亚型呈现肺鳞癌、肺小细胞癌所占比例逐渐下降，肺腺癌则逐渐上升趋势，与世界其他国家相似。本研究利用2000年-2012年中国医学科学院肿瘤医院的男性肺癌诊治病例，探讨肺癌患者不同吸烟状态下组织学亚型的特征及变化趋势。

**1:** 肺癌患者基本情况 Characteristics of lung cancer patients

Items	Smoking			Non-smoking		All	*X*^2^	*P*
	*n*	%		*n*	%	(*n*)		
Total	11, 750	83.30		2, 356	16.70	14, 106		
Age groups (yr)							218.52	< 0.001
< 30	32	52.46		29	47.54	61		
30-39	307	66.45		155	33.55	462		
40-49	1, 726	79.58		443	20.42	2, 169		
50-59	3, 798	86.59		588	13.41	4, 386		
60-69	3, 960	85.33		681	14.67	4, 641		
70-79	1, 814	80.98		426	19.02	2, 240		
80+	113	76.87		34	23.13	147		
Year of diagnosis							17.81	0.12
2000	387	84.50		71	15.50	458		
2001	423	83.76		82	16.24	505		
2002	541	84.40		100	15.60	641		
2003	503	83.42		100	16.58	603		
2004	646	82.08		141	17.92	787		
2005	835	80.60		201	19.40	1, 036			
2006	921	83.65		180	16.35	1, 101		
2007	1, 036	84.71		187	15.29	1, 223		
2008	1, 088	84.02		207	15.98	1, 295		
2009	1, 211	84.98		214	15.02	1, 425		
2010	1, 402	83.85		270	16.15	1, 672		
2011	1, 512	82.67		317	17.33	1, 829		
2012	1, 245	81.32		286	18.68	1, 531		
Histology							656.88	< 0.001
SCC	4, 627	92.19		392	7.81	5, 019		
ADC	3, 507	73.43		1269	26.57	4, 776		
SCLC	1, 828	86.96		274	13.04	2, 102		
LCC	428	83.76		83	16.24	511		
ASC	233	84.42		43	15.58	276		
Other	1, 127	79.25		295	20.75	1, 422		
Residents' province							10.90	0.053
Beijing	3, 523	82.95		724	17.05	4, 247		
Hebei	1, 937	84.03		368	15.97	2, 305		
Inner Mongolia	1, 071	85.00		189	15.00	1, 260		
Shandong	786	81.54		178	18.46	964		
Heilongjiang	704	80.64		169	19.36	873		
Other	3, 729	83.67		728	16.33	1, 715		
SCC: squamous cell carcinoma; ADC: adenocarcinoma; SCLC: small cell lung cancer; LCC: large cell carcinoma; ASC: adenosquamous carcinoma.								

**1:**
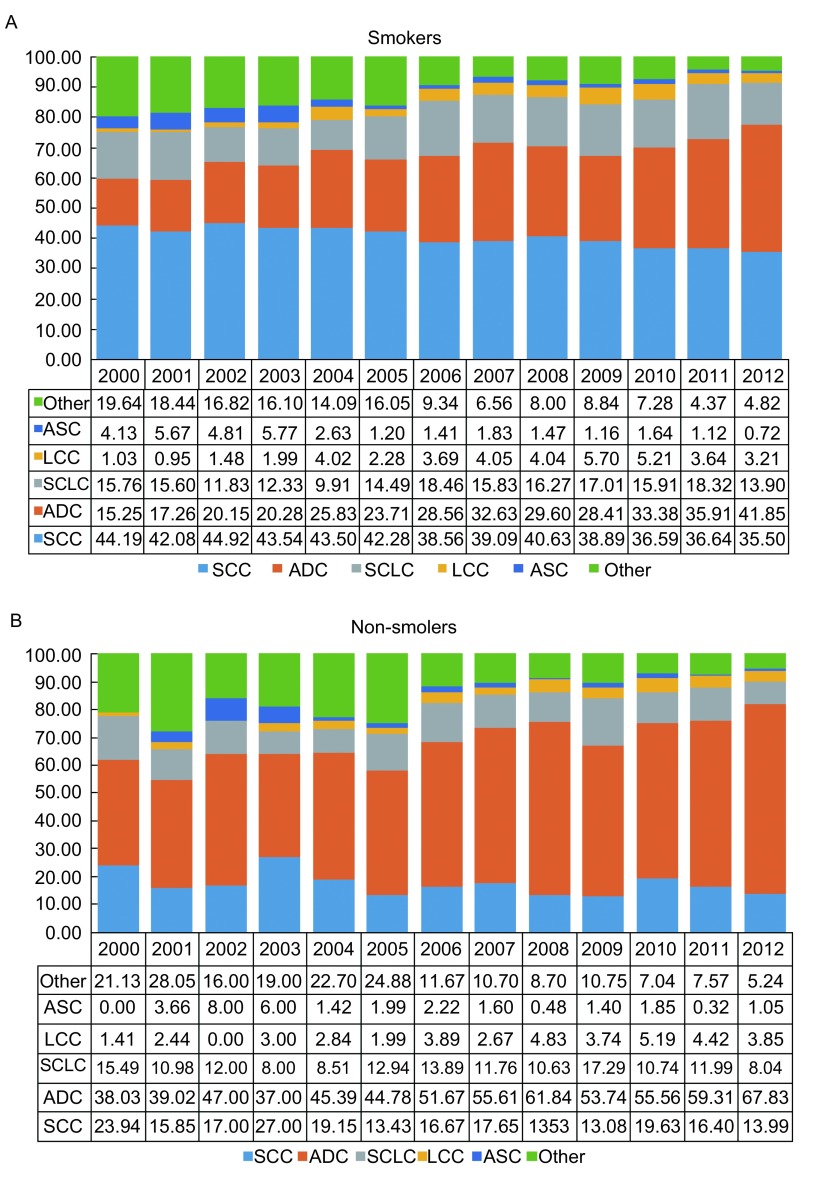
肺癌吸烟患者和不吸烟患者的组织学分型的变化。2000年-2012年吸烟者肺癌组织学类型变化趋势（annual percentage change, APC）：A:鳞癌：APC=-1.9%，*P* < 0.001；腺癌：APC=6.8%，*P* < 0.001；小细胞癌：APC=1.5，*P*=0.23；大细胞癌：2000年-2009年：APC=15%，*P*=0.01；2009年-2012年：APC=-16.2%，*P* =0.09；腺鳞癌：APC=-14.9%，*P* < 0.001；其他类型：APC=-10.6，*P* < 0.001；B：鳞癌：APC=-3.0%，*P*=0.07；腺癌：APC=4.3%，*P* < 0.001；小细胞癌：APC=-0.6%，*P*=0.78；其他类型：APC=-10.7%，*P* < 0.001；大细胞癌和腺鳞癌呈波动变化. Distribution and trend of histology in smoking and non-smoking lung cancer patients.. APC in smoking lung cancer patients f rom 20 0 0 to 2012: A: SCC: APC= -1.9 %, *P* < 0.001; ADC: APC=6.8%, *P* < 0.001; SCLC: APC=1.5, *P*=0.23; LCC: 2000-2009: APC=15%, *P*=0.01, 2009-2012: APC=-16.2%, *P*=0.09; ASC: APC=-14.9%, *P* < 0.001; Other: APC=-10.6, *P* < 0.001; B: SCC: APC=-3.0%, *P* =0.07; ADC: APC=4.3%, *P* < 0.001; SCLC: APC=-0.6%, *P*=0.78; Other: APC=-10.7%, *P* < 0.001; Distributions of LCC and ASC were fluctuating.

吸烟的肺癌患者中鳞癌所占比例最高，为39.38%，不吸烟患者中肺腺癌比例最高，为53.86%，与其他研究^[12-14]^相似。肺癌吸烟≥20包年、 < 10包年和10包年-20包年患者中，鳞癌差异较大（分别为44.41%、32.21%、31.93%），提示吸烟与肺鳞癌发生具有剂量效应反应关系。吸烟病例中鳞癌和腺鳞癌比例均呈显著下降趋势（APC=-1.9%, -14.9%）；腺癌每年以6.8%速度上升（*P* < 0.001）。多项文献^[7, 17]^报道，这可能与过滤卷烟使烟草烟雾的成分发生了改变相关。过滤香烟的使用理论上可减少尼古丁、焦油和一氧化碳的含量，但由于吸烟者的补偿行为如堵住滤嘴上透气孔、加大吸入烟草烟雾量等，并没有减少吸烟者体内的尼古丁和焦油含量，而加大烟草烟雾量和过滤嘴的使用，使小成分烟草烟雾更易到达肺外周气道，进而造成腺癌高发。也有文献^[18]^报道助燃剂的使用使一氧化氮增加，促进亚硝胺类物质的形成，而亚硝胺-4-（甲基化亚硝胺类）-1-（3-吡啶基）-1-丁酮与肺腺癌密切相关。本研究对吸烟剂量分层发现，鳞癌在吸烟 < 10包年中的下降速度最快，腺癌的上升速度则在≥20包年中最快，提示肺癌患者中腺癌和鳞癌的改变受吸烟剂量的影响。

在不吸烟的肺癌患者中也观察到腺癌比例显著上升（APC=4.3%, *P* < 0.001）。是否与空气污染日益严重相关，应进一步研究。细颗粒物PM2.5为是空气污染物中的重要组成成分，可深入到人体细支气管和肺泡。已有研究^[19]^证明，PM2.5可刺激肺部细胞一氧化氮的表达。也有报道^[20]^称非吸烟肺癌患者中腺癌的下降可能与被动吸烟、烹饪油烟、环境职业暴露等相关。不吸烟病例中也观察到鳞癌比例的下降，但该下降并不显著。

**2:** 吸烟包年与肺癌病理组织分型构成比（%）及变化趋势 Distribution and trend of histology in diffident smoking pack-years of lung cancer patients

Pack-years	Total	Year of diagnosis													APC
		2000	2001	2002	2003	2004	2005	2006	2007	2008	2009	2010	2011	2012	
SCC															
< 10	32.21	42.50	32.35	41.36	39.73	34.48	32.97	29.00	35.28	36.67	31.36	28.90	27.06	25.34	-3.3^*^
10-19	31.93	20.69	34.48	39.47	37.14	42.00	39.13	26.76	32.84	31.88	31.94	35.71	24.27	26.97	-2.8
≥20	44.41	47.90	48.06	47.76	45.96	47.39	47.96	45.27	42.88	44.66	44.66	41.68	42.84	41.11	-1.3^*^
ADC															
< 10	32.31	15.00	20.59	24.61	21.23	27.01	26.09	31.33	36.92	28.25	31.14	36.80	37.42	46.83	6.5^*^
10-19	39.13	24.14	24.14	15.79	14.29	44.00	26.09	46.48	32.84	47.83	36.11	42.86	54.37	49.44	4.8^*^
≥20	27.34	14.29	14.73	17.95	20.50	23.22	22.04	24.73	29.21	28.45	25.77	30.16	33.12	38.71	7.1^*^
SCLC															
< 10	18.88	13.33	19.12	10.99	12.33	14.94	15.58	23.67	15.42	20.27	19.52	20.23	24.74	19.83	3.6^*^
10-19	13.54	27.59	13.79	7.89	14.29	6.00	20.29	9.86	17.91	8.70	16.67	14.29	13.59	10.11	-5.0^*^
≥20	13.86	15.55	13.95	12.82	12.11	8.29	13.06	16.73	15.90	14.14	15.37	13.27	15.60	11.60	0.2
*: *P* < 0.05.															

肺癌吸烟患者中，大细胞癌在2009年以前显著上升，2009年后下降；不吸烟患者中2010年后呈下降趋势，可能与组织学诊断技术的提升有关，如腺状或鳞状分化免疫标记物的在组织学诊断中的应用使腺癌、鳞癌的诊断更为明确，2015版世界卫生组织肺癌组织学分类中明确指出，如果TTF-1或p40阳性，显示实性生长方式的肿瘤应分别被重新归类为实性型腺癌或非角化型鳞状细胞癌，而非大细胞癌^[21]^。其他组织学类型在吸烟和不吸烟肺癌患者中均显著下降，这可能由于组织学诊断技术的提高使一些不能诊断或不明诊断类型比例下降。

肺癌的发病和死亡均居恶性肿瘤首位，为我国居民带来严重的疾病负担。男性吸烟率居高不下，低焦油和过滤卷烟的低认知^[8]^，给控制烟草的流行带来难度，控制烟草仍是今后肺癌防治的重中之重。本研究发现，腺癌在肺癌吸烟和不吸烟病例中所占的比例均显著上升，鳞癌在肺癌吸烟病例中的比例显著下降，该趋势受吸烟剂量的影响，为加强肺癌防治有一定的参考意义。
